# Spontaneous small bowel perforation: a rare GI manifestation of type IV Ehlers-Danlos syndrome

**DOI:** 10.1093/jscr/rjac192

**Published:** 2022-04-30

**Authors:** Alexandria M Gallagher, Tara L McGraw, Fredrick K Toy

**Affiliations:** 1 Department of General Surgery, Geisinger Wyoming Valley Hospital, Wilkes-Barre, PA, USA; 2 Department of Trauma and Emergency General Surgery, Geisinger Wyoming Valley Hospital, Wilkes-Barre, PA, USA

## Abstract

Isolated small bowel perforation is low in the differential diagnosis of abdominal pain in the young, relatively healthy patient. It is, however, a rare manifestation of type IV (vascular) Ehlers-Danlos syndrome (EDS). In addition, there is no general consensus on the management of GI manifestations in patients with type IV EDS. We present the case of a 31-year-old male with history of type IV EDS, presenting with acute onset abdominal pain. Imaging was notable for intra-abdominal free air and thickened loops of small bowel in the pelvis. The patient underwent exploratory laparotomy with resection of the small bowel perforation with enteroenteric anastomosis. In our literature review, we evaluated gastrointestinal manifestations observed in patients with type IV EDS, management recommendations and potential complications to be mindful of in this population.

## INTRODUCTION

Spontaneous small bowel perforation is a rare differential in the young patient with acute abdominal pain. This manifestation can be seen in patients with type IV vascular Ehlers-Danlos (EDS IV, vEDS) which is associated with arterial, digestive and uterine complications, which are rarely seen in the other types of Ehlers Danlos. Of all patients with EDS, type IV represents 5–10% of cases [1].

## CASE REPORT

**Figure 1:**
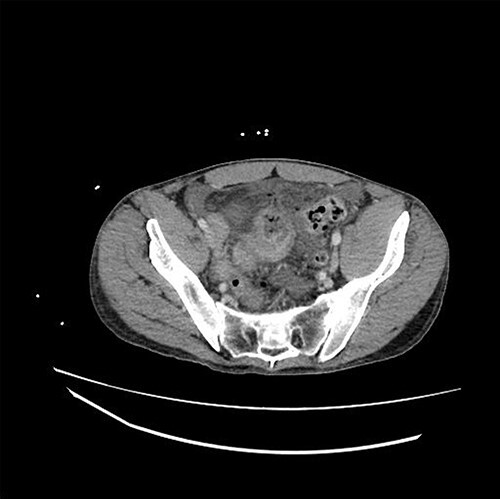
CT demonstrating thickened loop of ileum in the pelvis with pneumoperitoneum and free fluid.

The patient is a 31-year-old male with medical history significant for EDS Type IV who presented for evaluation of sudden onset of abdominal pain, which woke him from sleep. He had a medical history significant of mitral valve prolapse and prior bacterial endocarditis resulting in bovine pericardial mitral valve replacement (MVR) on Coumadin, hemorrhagic left parietal stroke, history of ventilator dependent respiratory failure s/p tracheostomy and percutaneous endoscopic gastrostomy tube (PEG) with subsequent decannulation and removal. Current medications included aspirin 81 mg, warfarin, furosemide, Lisinopril, metoprolol and fluoxetine. Admission vital signs were unremarkable. Physical exam revealed a distended, diffusely tender abdomen with involuntary guarding. The lab values were significant of a leukocytosis of 18.53, INR 2.50 and lactic acid of 2.7. A computed tomography scan of the abdomen and pelvis with intravenous contrast demonstrated a dilated loop of distal ileum with associated mucosal thickening, free air and free fluid concerning for bowel perforation (Fig. 1).

The patient was admitted to the intensive care unit and emergently taken to the operating room for exploratory laparotomy. Upon entering the abdomen, there was noted to be purulent peritonitis and a culture was taken. There was a loop of ileum with a fibrinous peel adherent to the pelvic side wall which was found to have a small necrotic area and succus was expressed. A small bowel resection was performed with an end-to-end, side-to-side functional enteroenteric anastomosis.

**Figure 2:**
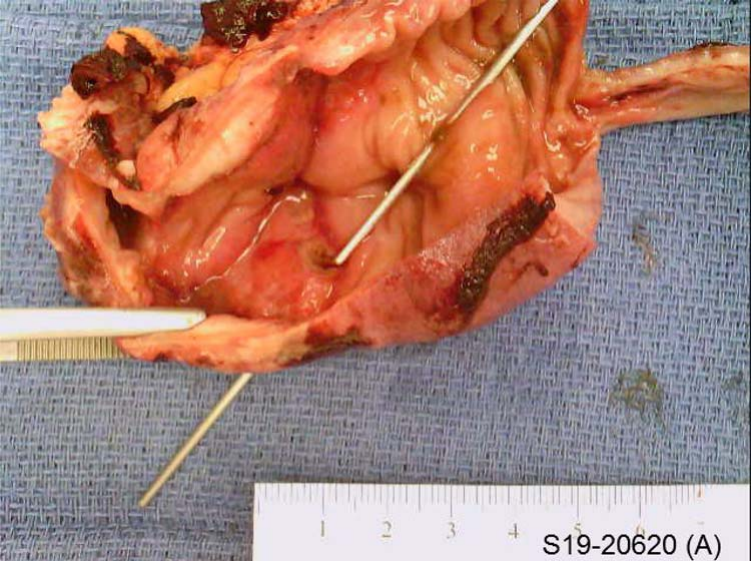
Gross pathology specimen with probe depicting area of perforation.

**Figure 3:**
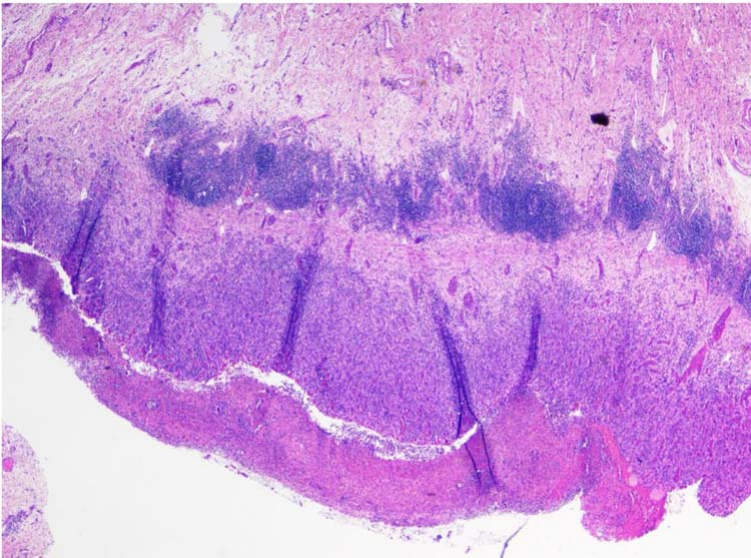
Histologic evaluation of specimen showing ulcerated small bowel wall with transmural and subserosal acute inflammatory changes.

He progressed well post-operatively. As he regained bowel function, his diet was slowly advanced and he was discharged home on post-op Day 7. Surgical pathology revealed gross perforation, fibrinous serosal exudate contiguous with perforated region and an area of partially circumferential stricture at the site of resection (Fig. 2). Microscopic examination noted transmural and subserosal exuberant acute inflammatory changes with areas of ulcerated small bowel (Fig. 3).

## DISCUSSION

EDS are a heterogeneous group of hereditary disorders of connective tissue with prevalence between 1 in 10 000 and 1 in 25 000 [2]. Hippocrates first described the hypermobility associated with EDS in 400 BC. The syndromes are named after two physicians, Evard Ehlers, a Danish dermatologist in 1901 [3] and Henri-Alexandre Danlos, a French dermatologist in 1908 [4].

Classification of this clinically and genetically heterogeneous group of conditions began in the late 1960s. In 1985, the Berlin Classification was established with two subtypes. This was further refined in 1998 as the Villefranche classification into six subtypes designated as roman numerals [5].

Our patient presented with known EDS type IV, the vascular type. This results from mutations in the gene for type III procollagen (COL3Ar). This makes up $\sim$5–10% of EDS. This mutation results in quantitative and qualitative abnormalities of mature type III collagen [2, 6]. Systemic arteries that are rich in type III collagen may undergo dissection, aneurysms or rupture. In addition to vascular complications, rupture of hollow viscera that is rich in type III collagen such as intestines, especially the sigmoid colon, and the uterus, is a potential complication of the disease. Pneumothorax is also a common complication since the pleura contains a high level of type III collagen. The clinical diagnosis of vascular EDS rests on the finding of at least two of four diagnostic criteria (thin translucent skin; arterial, intestinal or uterine rupture; easy bruising; and characteristic facial appearance), but laboratory studies are necessary for confirmation. These would include skin biopsy and genetic testing [6, 7].

There is no known cause for EDS and treatment is supportive. Monitoring the cardiovascular system, physical and occupational therapy, acquatic therapy, and orthotics (bracing) are supportive measures that can improve quality of life [8]. If surgery is necessary for anything, it requires careful tissue handling, longer wound healing and longer immobilization post-operatively [9, 10]. Most EDS patients live a normal lifespan. The blood vessel fragility in vascular EDS patients, however, leads to a high complication rate, with arterial rupture being the most common cause of sudden death (75%) than gastrointestinal rupture (8%). The median life expectancy with vEDS is 48 years [6, 7]. It is imperative when caring for vascular EDS patients who present with acute onset abdominal pain that spontaneous perforation is within the differential to ensure timely intervention and treatment.
